# Bacterial Toxin Fusion Proteins Elicit Mucosal Immunity against a Foot-and-Mouth Disease Virus Antigen When Administered Intranasally to Guinea Pigs

**DOI:** 10.1155/2011/713769

**Published:** 2011-09-22

**Authors:** Sreerupa Challa, Steven M. Szczepanek, Debra Rood, Roger W. Barrette, Lawrence K. Silbart

**Affiliations:** ^1^Department of Animal Science, University of Connecticut, Storrs, CT 06269, USA; ^2^Center of Excellence for Vaccine Research, University of Connecticut, Storrs, CT 06269-2191, USA; ^3^Department of Biomedical Sciences, Division of Infectious Diseases, Tufts Cummings School of Veterinary Medicine, North Grafton, MA 01536, USA; ^4^Department of Allied Health Sciences, University of Connecticut, Storrs, CT 06269, USA; ^5^Plum Island Animal Disease Center, USDA-APHIS/VS/FADDL, Greenport, NY 11944, USA

## Abstract

Peptides corresponding to the foot-and-mouth disease virus VP1 G-H loop are capable of inducing neutralizing antibodies in some species but are considered relatively poor immunogens, especially at mucosal surfaces. However, intranasal administration of antigens along with the appropriate delivery vehicle/adjuvant has been shown to induce mucosal immune responses, and bacterial enterotoxins have long been known to be effective in this regard. In the current study, two different carrier/adjuvant approaches were used to augment mucosal immunity to the FMDV O_1_ BFS G-H loop epitope, in which the G-H loop was genetically coupled to the *E. coli* LT-B subunit and coexpressed with the LTA2 fragment (LTA2B-GH), or the nontoxic pseudomonas exotoxin A (ntPE) was fused to LTA2B-GH at LT-A2 to enhance receptor targeting. Only guinea pigs that were inoculated intranasally with ntPE-LTA2B-GH and LTA2B-GH induced significant anti-G-H loop IgA antibodies in nasal washes at weeks 4 and 6 when compared to ovalbumin or G-H loop immunized animals. These were also the only groups that exhibited G-H loop-specific antigen-secreting cells in the nasal mucosa. These data demonstrate that fusion of nonreplicating antigens to LTA2B and ntPE-LTA2B has the potential to be used as carriers/adjuvants to induce mucosal immune responses against infectious diseases.

## 1. Introduction

The generation of an effective mucosal immune response to foreign proteins often requires the addition of a carrier/adjuvant molecules, many of which are bacterial exotoxins such as cholera toxin (CT), heat-labile enterotoxin (LT), pertussis toxin (PT), and exotoxin A (PE) secreted by *Vibrio cholera, Escherichia coli, Bordetella pertussis,* and *Pseudomonas aeruginosa,* respectively. Each of these toxins possess ADP-ribosylating activity, and their nontoxic forms can be used as mucosal carrier adjuvants because of their ability to bind to receptors on the epithelial cell surface, thereby, facilitating their delivery to the underlying lymphoepithelial tissue [1]. Moreover, antitoxin responses are so potent that they generate strong immune responses against foreign molecules that are simultaneously present at the mucosal surfaces through a bystander effect. It has also been shown that coadministration of foreign proteins with potent mucosal adjuvants can prevent the induction of oral tolerance [2]. Regrettably, widespread use of toxin-based mucosal adjuvants has been dramatically restricted due to the inherent toxicity of these agents [3], necessitating the development of less effective “toxoids.” 

CT and LT, the most extensively studied mucosal adjuvants in animal models to date, belong to the AB_5_ class of bacterial toxins composed of a receptor-binding pentameric B subunit and an enzymatically active A subunit. The B subunit (LT-B) is a 103-amino acid protein that self-assembles into a 55 kDa pentameric structure that is responsible for binding to various eukaryotic cell receptors [4]. LT-B has been found to be associated with multiple functions, including receptor binding and the ability to induce apoptosis of CD8+ [5] (and occasionally CD4+) T cells. The A subunit (LT-A) is noncovalently linked to LT-B by a trypsin-sensitive loop and an *α*-helical region that joins the two fragments [6]. LT-A has two subunits (A1 & A2) linked by a disulfide bond, wherein the globular A1 subunit is enzymatically active and the C-terminal A2 subunit is anchored into the pentameric B subunit [7]. The A1 subunit contains an ADP-ribosylation site that binds to NAD^+^ and transfers ADP ribose to the alpha subunit of GTP-binding proteins involved in signal transduction. This leads to the activation of adenylate cyclase and abnormal accumulation of intracellular cAMP, ultimately leading to fluid and electrolyte efflux from the cell, and the watery diarrhea observed in the host [8]. The A1 subunits of CT and LT were also found to bind ADP ribosylation factors (ARFs) that are involved in the enhancement of ADP-ribosylating activity [9]. Genetic fusion of foreign antigens to the A2 portion of AB_5_ enterotoxins (of CT and LT), in place of the A1, catalytic subunit has been shown to be potent mucosal adjuvants and carrier proteins. Such fusion proteins have been used to elicit mucosal immune responses against the SBR from the streptococcal adhesin AgI/II [10], the gonococcal transferring-binding proteins TbpA and TbpB [11], and serine-rich *Entamoeba histolytica* protein (SREHP, fused to a maltose-binding protein (MBP)) [12]. 

PE is a single-unit bacterial exotoxin which exhibits NAD^+^-diphthamide ADP-ribosyl transferase activity, and binding of PE to its receptor (*α2*-macroglobulin receptor/low density lipoprotein receptor-related protein (LRP1)/CD91) initiates receptor-mediated endocytosis followed by the exertion of its toxic effects [13–16]. Fusion of an inactivated form of this toxin (ntPE) with the V3 loop of HIV has been shown to induce mucosal immune responses *in vivo* [17, 18], indicating that such an approach may be useful for vaccine design directed against mucosal pathogens. Previous studies have indicated that the intranasal administration of foot and mouth disease virus (FMDV) O_1_ BFS G-H loop peptides do not induce protective immune responses in cattle [19]; however, the ntPE-GH fusion protein induced anti-G-H serum IgG antibodies along with anti-ntPE serum IgG and mucosal IgA antibodies when intranasally administered to pigs [20], indicating that fusion proteins coupled to G-H loop peptides could make useful mucosal vaccines. 

The central hypothesis of this work is that an antigen, such as the FMDV G-H loop, will induce respiratory mucosal immune responses against the epitope when genetically coupled to the C-terminus of LT-B (where the toxic LT-A1 domain is replaced with the mucosal adjuvant ntPE) and delivered to animals through the nasal route. This hypothesis is based on the observations that (1) a consensus G-H loop peptide (administered parenterally) induced protection in pigs upon virus challenge [21], (2) mucosal immunization of pigs through the nasal route has been shown to induce both systemic IgG and nasal IgA antibodies [20], and (3) the G-H loop antigen, when coupled to the mucosal adjuvant ntPE, induced a modest immune response against the G-H loop epitope when administered intranasally to pigs [20]. 

 In the present study, we constructed the chimeric proteins LTA2B-GH and ntPE-LTA2B-GH by inserting the coding sequence of the FMDV O_1_ BFS G-H loop onto the C-terminus of LT-B. Inserting the G-H loop onto LT-B allows five copies of the antigen to be displayed to the host's immune system as LT-B pentamerizes while fusing ntPE in place of the toxic A1 moiety will allow additional receptor targeting properties of the fusion protein. Both fusion proteins were evaluated for antigenic display of the G-H loop and pentamerization of the LT-B subunit. We then evaluated the mucosal immunogenicity of these two nontoxic chimeric proteins against the FMDV G-H loop in guinea pigs and found that they are capable of inducing antigen specific secretory IgA immune responses in the respiratory tract of immunized animals.

## 2. Materials and Methods

### 2.1. Plasmid Construction

The LT-A2/LT-B (referred to as LTA2B) fragment from the plasmid *pEnt *[22] was PCR amplified and cloned into an N-terminus His_6_ tag containing expression vector, *pET-28 b(+) *(Novagen, EMD, Gibbstown, NJ), between restriction enzyme sites *BamH1 *and *Sal1* using the following primers: LTA2B-BamH1-F.P: GCC CGT TGC *GGA TCC* G GGT GAT ACT TGT AAT GAG GAG and LTA2B- Sal1-R.P: GCG AGC CGA *GTC GAC *GTT TTT CAT ACT GAT TGC CGC. This resulted in the generation of the plasmid *pET-LTA2B*. The G-H loop sequence from the previously generated ntPE-GH construct [20] was amplified by PCR and subcloned into *pET-LTA2B *at the 3′ end of the LTA2B gene, between restriction enzyme sites *Sal1 *and *Xho1* using the following primers: GH-Sal1-F.P: GCC CGT TGC *GTC GAC* CGT TAT AGT AGA AAC GCG GTG and GH-Xho1-R.P: GCG AGC CGA *CTC GAG *CAG TGT GCG AGC AAC TTT CTG. This resulted in the generation of the plasmid *pET-LTA2B-GH*. ntPE from pIVEX-ntPE [20] was PCR amplified and cloned into a N-terminus His_6_ tag containing expression vector, *pET-28 b(+), *between restriction enzyme sites *Nde1 *and *HindIII *using the following primers: ntPE-Nde1-FP: GCC CGT TGC *CAT ATG* GCC GAG GAA GCC TTC GAC CTC and ntPE-HindIII-RP: GCG AGC AGA *AAG CTT *CTT CAG GTC CTC GCG CGG CGG. This resulted in the generation of the plasmid *pET-ntPE*. LTA2B-GH from the construct pET-LTA2BGH was PCR amplified and sub-cloned into *pET-ntPE *at 3′ terminus of the ntPE gene between the restriction enzyme sites *HindIII *and *Not1* using the following primers: LTA2BGH-HindIII-F.P: GCC CGT TGT *AAG CTT* GGT GAT ACT TGT AAT GAG GAG and LTA2BGH-Not1-R.P: GCG AGC CGA C*GC GGC CGC* CAG TGT GCG AGC AAC TTT CTG. This resulted in the generation of the plasmid *pET-ntPE-LTA2B-GH*. 

### 2.2. PCR

For each of the above experiments, 50 *μ*L PCR reaction mixture was made by using 5% DMSO, 1 *μ*M of each primer, 200 *μ*M of each nucleotide, 100 ng DNA, 1X *Pfu *Turbo DNA Polymerase reaction buffer, and 2.5 U of *Pfu *Turbo DNA Polymerase (Stratagene, La Jolla, Calif, USA). The PCR reaction was carried out as follows: 94°C for 2 min, followed by 30 cycles of 94°C for 30 sec, 65°C for 30 sec, 72°C for 2 min, and finally followed by elongated extension at 72°C for 7 min.

### 2.3. Expression of Fusion Proteins

The constructs, *pET-LTA2B-GH* and *pET-ntPE-LTA2B-GH,* were transformed into Rosetta 2 (DE3) pLysS cells (Novagen) and plated on 50 *μ*g/mL kanamycin and 34 *μ*g/mL chloramphenicol containing Lysogeny broth (LB) agar plates. Positive clones were cultured in 25 mL LB media containing 50 *μ*g/mL kanamycin and 34 *μ*g/mL chloramphenicol for 16 hr at 37°C in a shaker incubator. Cultures were then transferred to 475 mL LB media containing 50 *μ*g/mL kanamycin and incubated for another 4 hr on a shaker incubator until the optical density at 280 nm (OD_280_) of the cultures reached 0.5-0.6. Later, protein synthesis was induced upon the addition of 1 mM IPTG, and cultures were incubated for 4 hr at 37°C in a shaker incubator. The cells were centrifuged at 2600 × g for 10 min, and the cell pellets were stored at −20°C overnight. The pellets were then thawed, after which 12 mL of Cellytic B reagent *(Sigma-Aldrich, St. Louis, Mo, USA)* and 20 *μ*L benzonase nuclease *(Novagen)* were added and vortexed until the samples were no longer viscous, and then incubated at room temperature (RT) for 10 min. Solutions were then transferred to 35 mL centrifuge tubes, and the inclusion bodies were pelleted by centrifuging at 23,360 × g for 10 min using the Sorvall super T21 tabletop centrifuge. The soluble fractions (supernatant) were saved, and inclusion bodies were resuspended in 6 mL of Cellytic B, and 300 *μ*L of 10 mg/mL lysozyme solution *(Sigma) *was subsequently added, vortexed, and incubated at RT for 10 min. Following incubation, 10 mL of 1 : 10 Cellytic B was added and centrifuged at 16,000 × g for 10 min. The supernatant was discarded, and inclusion bodies were washed with an additional 10 mL of 1 : 10 Cellytic B. The washes were repeated three times, after which the inclusion bodies were solubilized in 10 mL of urea buffer (8 M urea in 50 mM sodium phosphate, 0.3 M NaCl buffer at pH 8.0).

### 2.4. Purification of Fusion Proteins

His-Select Ni^+2^ resin *(2.0 mL; Sigma)* was centrifuged in a 15 mL Falcon tube at 200 × g for 5 min to remove ethanol. The resin was washed once with 13 mL dd H_2_O and equilibrated using 13 mL of urea buffer. The resin was then incubated with solubilized inclusion bodies at RT for 1 hr followed by centrifugation at 200 × g for 5 min, and the supernatant was discarded. The resin was washed twice with 13 mL of urea buffer and the purified protein eluted by adding 10 mL of elution buffer (10 M urea, 150 mM imidazole) and incubated for 30 min at RT on a shaker.

### 2.5. SDS-PAGE

Samples were mixed with 2x Laemmli Sample buffer *(Sigma)* at a 1 : 1 ratio and boiled for 10 min in a water bath prior to electrophoresis on 4–20% acrylamide gradient Ready Gel Tris-HCL *(BIO-RAD)* using SDS-running buffer (25 mM Tris, 192 mM glycine, 0.1% (w/v) SDS, pH 8.3). Gels were stained with Bio-safe coomassie stain *(BIO-RAD), *and then destained with distilled water to visualize proteins.

### 2.6. Western Blot

Proteins were transferred from polyacrylamide gels onto a polyvinylidene difluoride (PVDF) membrane in transfer buffer (25 mM Tris, 192 mM Glycine, 20% methanol, pH 8.3) for 1 hr at RT using Mini-Trans Blot system *(Bio-Rad, Hercules, Calif, USA)*. The membrane was then blocked for 1 hr at RT using 3% nonfat dry milk in PBS, supplemented with 0.05% Tween 20 (PBST), washed 3 times with PBST and probed with a 1 : 1,000 dilution of pig anti-G-H serum (hyperimmune antisera from pigs immunized with the FMDV type O consensus G-H loop containing peptide UBITh [21]) for 1 hr at RT followed by washing 5 times with PBST. The membrane was then incubated for 1 hr at RT with horseradish peroxidase-conjugated (HRP) goat anti-pig IgG *(Bethyl laboratories, Montgomery, Tex, USA)* and washed 5 times with PBST, thereafter. Antibody binding was detected with 3,3′,5,5′-tetramethylbenzidine (TMB, substrate kit for peroxidase, *Vector laboratories, Burlingame, Calif, USA*) until sufficient staining was detected.

### 2.7. GM1 ELISA

96-well polystyrene Immulon-4 HBX microtiter plates *(Dynex Technologies, Chantilly, VA)* were coated with 50 *μ*L of 10 *μ*g/mL GM1 (monosialotetrahexosylganglioside, *Sigma*) or BSA (negative control), diluted in coating buffer (0.05 M Na_2_CO_3_ buffer, pH 9.6). Plates were then tightly wrapped in Parafilm *(Pechiney plastic packaging, Menasha, Wis, USA)*, incubated overnight at RT, then stored at 4°C until they were used. On the day of assay, plates were washed three times with 200 *μ*L of wash buffer (50 mM Tris, 0.14 M NaCl, 0.05% Tween 20, pH 8.0) using a Bio-Tek ELx-405 plate washer *(Winooski, Vt, USA)* and incubated with 100 *μ*L of blocking solution (50 mM Tris, 0.14 M NaCl, 1% BSA, pH 8.0) for 1 hr at RT followed by washing three times with wash buffer. Then 50 *μ*L of the proteins, LTA2B-GH, ntPE-LTA2B-GH, cholera toxin B subunit (CTB), TCA peptide (a 53-mer peptide comprised of three FMDV O_1_ BFS strain epitopes, including a known VP4 T helper cell epitope, VP1 site C, and VP1 site A epitopes, “TCA” refers to the directionality of epitopes), and ntPE (20 *μ*g/mL) was added to separate wells and incubated at RT for 2 hr. The plates were washed 5 times with wash buffer, followed by the addition of 1 : 1,000 dilution of rabbit antibody to cholera toxin *(Virostat, ME)*, 1 : 5,000 dilution of rabbit antibody to Pseudomonas exotoxin A *(Sigma)* or a 1 : 500 dilution of pig antibody to consensus G-H peptide, in different wells, followed by incubation at RT for 1 hr. The plates were then washed 5 times with wash buffer and a 1 : 10,000 dilution of either goat antipig IgG conjugated to HRP *(Bethyl laboratories) *or goat antirabbit IgG conjugated to HRP *(Bethyl laboratories)* was added and incubated at RT for 1 hr. A final wash (5 times) was performed with wash buffer followed by the addition of TMB substrate *(KPL, Inc, Gaithersburg, Md, USA) *and stopped by adding 2 N H_2_SO_4_. The final OD was obtained at 450 nm using a Bio-Tek EL-311 plate reader. The net anti-ntPE/TCA peptide/CTB responses for each sample were calculated by subtracting the mean OD of the BSA-coated wells from the mean OD of specific antigen-incubated wells.

### 2.8. Animals

Forty-eight, female, two-week-old, Duncan-Hartley guinea pigs *(Elm Hill Labs, Chelmsford, Mass, USA)* were randomly divided into eight groups of six, corresponding to the vaccination groups described in Table 1. The guinea pigs were housed in environmentally controlled rooms, and food and water were provided *ad libitum*. All animal procedures were conducted in accordance with an approved University of Connecticut IACUC protocol (No. A07-025).

### 2.9. Guinea Pig Vaccinations and Procedures

Guinea pigs were anaesthetized using aerosolized isoflurane *(Baxter Healthcare Co., Deerfield, Ill, USA)*, with doses calculated based on body weight. Groups were immunized with 100 *μ*g of protein immunogen via the intranasal (i.n.) or subcutaneous (s.c.) route, as indicated in Table 1. The immunogens tested included Ovalbumin (control), TCA peptide, LTA2B-GH, and ntPE-LTA2B-GH. The first four groups (I-IV) received i.n. vaccinations while the other groups (V-VIII) received s.c. vaccination with the MPL+TDM+CWS Adjuvant system *(Sigma)* in a 1 : 1 ratio. All animals were primed on day 0, and the i.n. vaccinated animals were boosted at weeks 1, 2, and 3 with the protein corresponding to their groups while the s.c. vaccinated animals received a booster dose only at week 3 (Table 1). Dosages of all vaccines (except for LTA2B-GH) were in a total volume of 200 *μ*L per guinea pig at 100 *μ*L/naris. The dosage of LTA2B-GH was administered dropwise in a total volume of 400 *μ*L per guinea pig at 200 *μ*L/naris. Sera and nasal washes were collected from animals at biweekly intervals. Nasal washes were collected by depositing, and then aspirating, 50 *μ*L of sterile PBS into each nostril. Blood was collected by cardiac puncture using 0.5 c.c. syringes and stored in heparin-treated tubes *(IRIS International Inc, Westwood, Mass, USA)* at RT for clotting, then centrifuging at 1500 × g for 10 min to obtain sera. Animals were sacrificed at week six while under sedation, upon intra-cardiac injection of Euthazol (1 mg/Kg, *Virbac AH Inc., Ft. Worth, Tex, USA*) prior to necropsy and tissue collection. 

### 2.10. Tissue Collection and Processing

The spleen and nasal mucosae were harvested from three of six animals from each group during necropsy. These tissues were washed in sterile RPMI 1640 + L-Glutamine *(CellGro, Mediatech Inc, Herndon, Va, USA)* and transferred to tubes containing complete RPMI media (RPMI 1640 + L-Glutamine + 10% FBS + 100 *μ*g/mL streptomycin sulfate, and 100 U/mL penicillin) and placed on ice. The spleens were disaggregated using a 10 mL syringe plunger in a Petri dish containing 10 mL of complete RPMI media, filtered through 10 mm nylon mesh, and centrifuged for 10 min at 4°C, 500 × g. To lyse RBCs, 5 mL of ACK lysis buffer (0.15 M NH_4_Cl, 10 mM KHCO_3_, 0.1 mM NaEDTA, pH 7.4) was added to the cell pellet, incubated for 5 min at RT, and centrifuged for 10 min at 4°C, 500 × g. The cell pellet was then resuspended in 5 mL of complete RPMI. The nasal mucosal tissue was washed three times in sterile media and then transferred into a 50 mL Erlenmeyer flask containing 20 mL of RPMI/EDTA (complete RPMI along with 5 mM EDTA, pH 8), and gently stirred for 15 min at room temperature. EDTA treatment/centrifugation was repeated two or three times, until the supernatant appeared clear. Residual EDTA was then removed by washing in complete RPMI media. Cells were then subjected to treatment with RPMI/collagenase (complete RPMI along with collagenase type 2 at 200 U/mL (Worthington Biochemical Corporation, Lakewood, NJ, USA)) for 1hr with gentle stirring at RT to release nasal mucosal lymphocytes. The contents of the flask were then filtered through a sterile 10 mm nylon screen, and the filtrate was centrifuged at 500 × g for 10 min at 4°C. The cell suspension was washed to remove residual collagenase, and the final pellet was resuspended in 5 mL of ice-cold RPMI-10. Both spleen and nasal mucosal lymphocytes were counted using a hemocytometer following trypan blue staining. Cells were then diluted to 10^6^ cells/mL in complete RPMI and held on ice until use. All steps were performed under aseptic conditions.

### 2.11. TCA Peptide/LTA2B-GH/ntPE-LTA2B-GH Specific ELISA

TCA peptide, LTA2B-GH and ntPE-LTA2B-GH specific IgG, IgM, and IgA antibodies in sera and nasal washes were assayed using an indirect ELISA. 96-well polystyrene Immulon-4 HBX microtiter plates *(Dynex Technologies)* were coated with 50 *μ*L of a 10 *μ*g/mL solution of either TCA peptide, LTA2B-GH, ntPE-LTA2B-GH, or BSA (negative control) in coating buffer (0.05 M Na_2_CO_3_ buffer, pH 9.6), tightly wrapped in Parafilm *(Pechiney plastic packaging)*, incubated overnight at RT and then stored at 4°C until needed. Plates were washed three times with 200 *μ*L of wash buffer (50 mM Tris, 0.14 M NaCl, 0.05% Tween 20, pH 8.0) using a Bio-Tek ELx-405 plate washer. Plates were then incubated with 100 *μ*L of blocking solution (50 mM Tris, 0.14 M NaCl, 1% BSA, pH 8.0) for 1 hr at RT followed by washing three times with wash buffer. Serum samples and nasal washes from weeks 0, 2, 4, and 6 were diluted at 1 : 50 and 1 : 4, respectively, in sample buffer (50 mM Tris, 0.14 M NaCl, 1% BSA, 0.05% Tween 20, pH 8.0) to determine sera IgG and nasal wash IgA antibodies. 50 *μ*L of each sample was applied in duplicate to the microtiter plate on all TCA peptide, LTA2B-GH, ntPE-LTA2B-GH and BSA-coated wells. After 1 hr of incubation at RT, the plates were washed five times with wash buffer, followed by the addition of goat antiguinea pig IgG antibody conjugated to HRP *(Bethyl laboratories)* (at a dilution of 1 : 5,000 in sample buffer) or rabbit anti-guinea pig IgA antibody (at a dilution of 1 : 1000 in sample buffer), and incubated for 1 hr at RT. Plates were then washed five times with wash buffer followed by the addition of goat antirabbit IgG antibody conjugated to HRP *(Bethyl laboratories),* diluted at 1 : 10,000 in sample buffer and incubated for 1 hr at RT. A final wash was performed, followed by the addition of TMB substrate *(KPL, Inc, Gaithersburg, Md, USA)* for 20 min at RT, and the addition of 2 N H_2_SO_4_ stop solution to terminate the reaction. The final OD reading was taken at 450 nm with an EL-311 Bio-Tek plate reader *(Winooski, Vt, USA).* The net anti-TCA Peptide/LTA2B-GH/ntPE-LTA2B-GH specific responses for each sample were calculated by subtracting the mean OD of the BSA-coated wells from the mean OD of specific antigen-coated wells.

### 2.12. ELISPOT

To detect IgG and IgA antibody secreting cells, Multiscreen-IP (0.45 *μ*M) 96-well plates *(Millipore, Bedford, Mass, USA)* were used. Plates were prewetted with 200 *μ*L/well of 70% ethanol and rinsed with sterile PBST. Plates were then coated with the following proteins: TCA peptide, LTA2B-GH and ntPE-LTA2B-GH and BSA control suspended in sterile coating buffer, and 50 *μ*L/well followed by incubation at 4°C overnight. Plates were then washed twice with sterile PBST (200 *μ*L per well) after bringing the plates to RT. Plates were then blocked with sterile blocking solution (100 *μ*L/well for 1 hr at RT) followed by washing the plate once with sterile PBST, 200 *μ*L/well. 10^6^ cells were then suspended in 200 *μ*L RPMI 1640 and added to designated wells and incubated in a 5% CO_2_ incubator at 37°C for 14–16 hr. Plates were then washed five times with PBST (200 *μ*L/well), followed by the addition of primary detecting antibody (goat-anti-guinea pig IgG HRP at 1 : 5,000 or rabbit antiguinea pig IgA at 1 : 5,000 dilutions in sample buffer), 50 *μ*L per well and incubation for 2 hr at RT. Rabbit-anti-guinea pig IgA-coated plates were washed five times with wash buffer, followed by the addition of goat antirabbit IgG antibody conjugated to HRP (*Bethyl laboratories,* diluted 1 : 10,000 in sample buffer and incubated for 1 hr at RT). Plates were then washed five times with PBST, and TMB substrate was added to develop the plates using a 3 : 2 ratio of TMB stock solution to hydrogen peroxide solution *(Vector Laboratories Inc.).* Spots were counted manually using a Lecia CME microscope *(Lecia Microsystems, Wetzlar, Germany)*.

## 3. Results

### 3.1. Generation, Expression, and Antigenicity of LTA2B-GH and ntPE-LTA2B-GH

**Figure 1:**
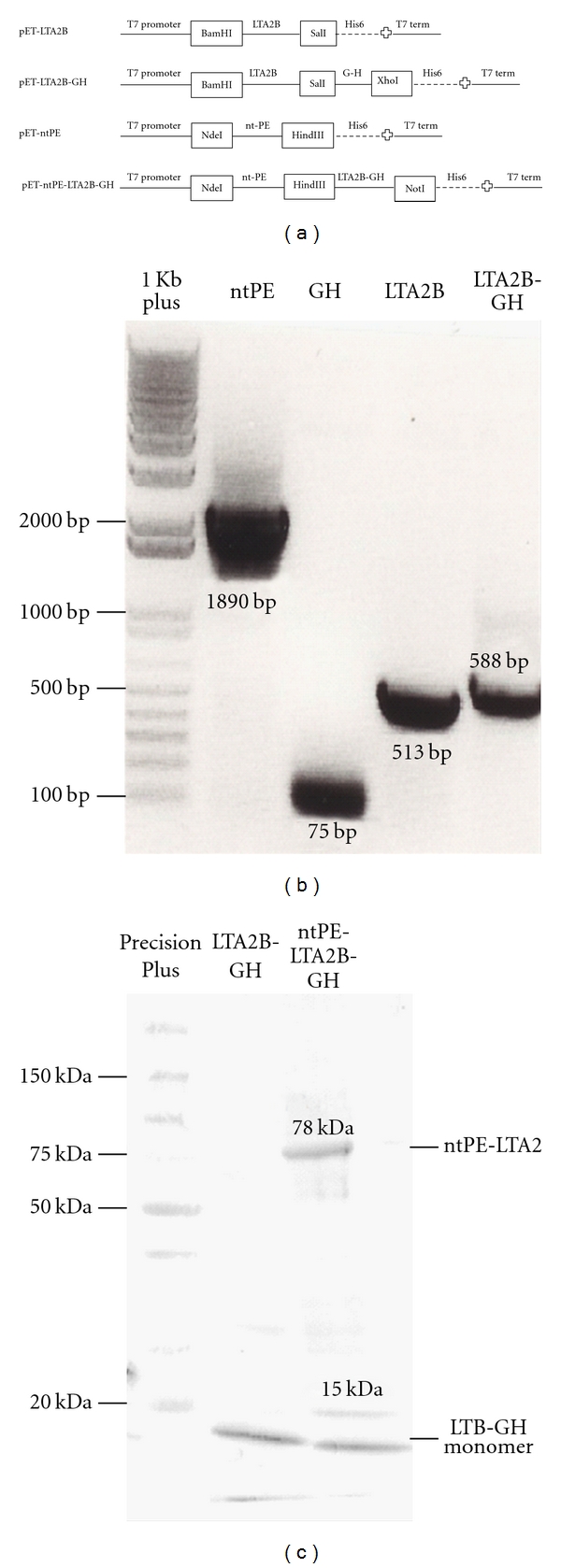
Generation and expression of fusion proteins. (a) Strategy for generation of recombinant fusion proteins by cloning amplicons from PCR reactions. (b) After construction a positive colony was selected (for each plasmid) and again screened for the presence of each fragments (ntPE, LTA2B, LTA2B-GH and the G-H loop) by PCR amplification. (c) Upon expression by IPTG and nickel column purification of proteins from *pET-LTA2B-GH, *and* pET-ntPE-LTA2B-GH*, western blots were performed using anti-G-H loop hyperimmune serum. Identified were proteins of approximately 15 and 78 kDa, respectively.

In this study, we generated, expressed, and purified the fusion proteins LTA2B-GH and ntPE-LTA2B-GH (which contain the mucosal adjuvants ntPE and LTA2B) coexpressed with the VP1 G-H loop of FMDV serotype O_1_ BFS as a model antigen for the elicitation of mucosal immune responses in guinea pigs (Figures 1(a) and 1(b)). The insertion of all sequences into expression plasmids was confirmed via screening colonies by PCR for the presence of ntPE (1890 bp), LTA2B (513 bp), G-H (75 bp), and LTA2B-GH (588 bp) (Figure 1(b)). All fusion proteins were highly expressed in *E. coli* BL21 (DE3) pLysS inclusion bodies after IPTG induction, as evidenced by thick bands in acrylamide gels (data not shown). The antigenicity of the G-H loop within the fusion proteins LTA2B-GH and ntPE-LTA2B-GH was confirmed by immunoblotting using hyperimmune antisera from pigs immunized with the FMDV consensus G-H loop peptide [21]. As anticipated, the G-H loop specific polyclonal antibodies recognized both LTA2B-GH and ntPE-LTA2B-GH (Figure 1(c)). Upon boiling in the presence of SDS, the B subunits of LTA2B-GH dissociated from each other, resulting in a single major band of approximately 15 kDa (monomeric form). The ntPE-LTA2B-GH revealed two major bands of the expected 78 kDa (LTA2-ntPE) and 15 kDa (LTA2B-GH monomer) size fragments.

### 3.2. GM1 ELISA

**Figure 2:**
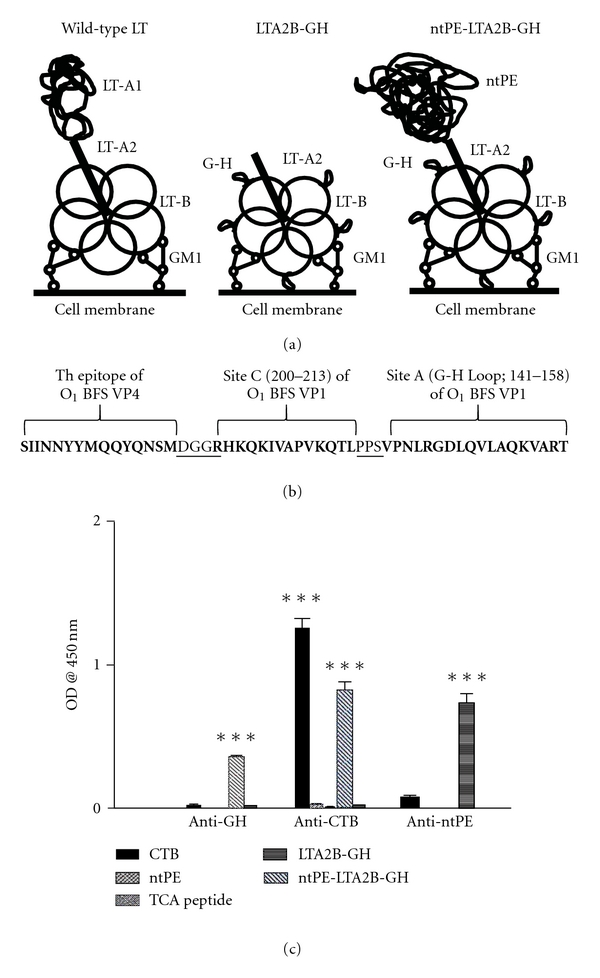
(a) Predicted structure of vaccine constructs. Wild-type LT binds to GM1 receptors upon pentamerization of LT-B and translocates the toxic LT-A subunit into host cells. LTA2B-GH is predicted to pentamerize in a similar fashion to the wild-type toxin, thereby, presenting five copies of the G-H loop to the host. ntPE-LTA2B-GH additionally contains ntPE in place of LT-A1 in order to expand receptor repertoire of the vaccine. (b) Amino acid sequence of the “TCA” peptide. The N-terminus consists of a promiscuous T-helper cells epitope from VP4 of FMDV (bold), followed by linking amino acids GG (underlined), “site C” from VP1 (bold), another linker sequence (PPS, underlined), and site A (the G-H loop). (c) GM1 ELISA to illustrate the pentamerization of LTA2B-GH and ntPE-LTA2B-GH. Microtiter plates were coated with GM1 and incubated with CTB, TCA peptide, ntPE, LTA2B-GH, and ntPE-LTA2B-GH. Then they were tested with anti-G-H, anti-CTB and anti-ntPE antibodies to illustrate the GM1-binding capacity of fusion proteins. ***P value < 0.001 according to ANOVA and post-hoc Tukey-Kramer multiple comparison test.

A GM1 ELISA was performed to assess binding capacity of LT-B-containing fusion proteins to this ganglioside (structures shown in Figures 2(a) and 2(b)). The TCA peptide and ntPE were not detected by the anti-GH loop antibodies as they have no mechanism for binding to GM1. Conversely, LTA2B-GH was immunoreactive with anti-CTB and anti-G-H antibodies, confirming that LTA2B-GH was properly folded and had pentamerized. Although ntPE-LTA2B-GH was not reactive to anti-CTB and anti-G-H antibodies, it did react with anti-ntPE antibodies, illustrating that the fusion protein (ntPE-LTA2B-GH) was able to bind to GM1 (Figure 2(c)), ostensibly as a result of the properly folded LTA2B-GH region of the fusion protein.

### 3.3. Antibody Responses to G-H Loop Peptide

**Figure 3:**
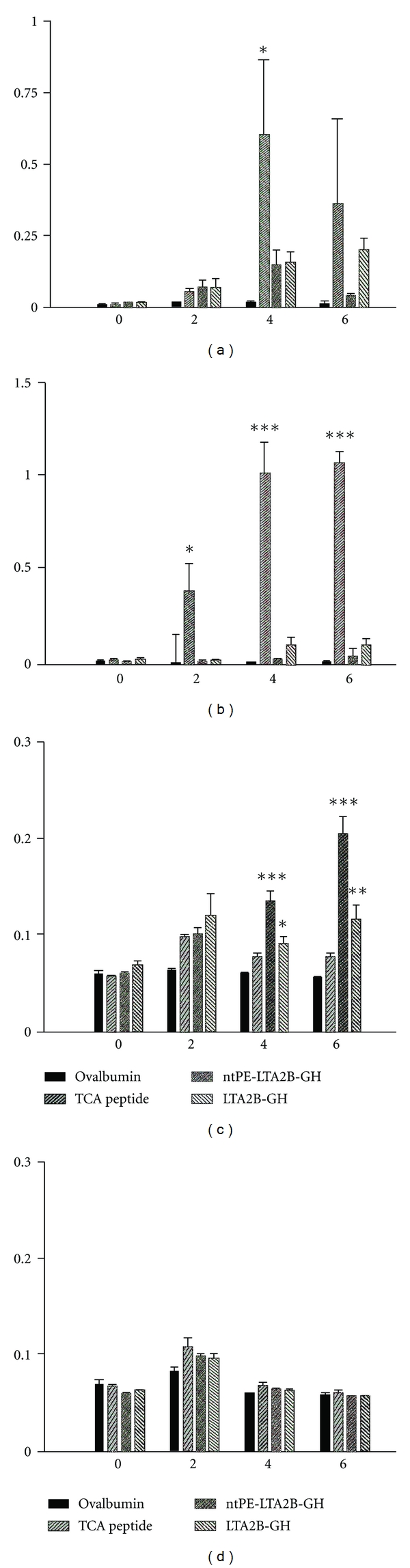
IgG and IgA antibody responses in serum and nasal wash samples. Pre- and postvaccination serum samples were collected every other week for six weeks from animals vaccinated i.n. (a) or s.c. (b) and were assayed for immunogen (left to right: ovalbumin, TCA peptide, ntPE-LTA2B-GH, LTA2B-GH) IgG responses by ELISA. Nasal washes were collected at the same time points and animals vaccinated i.n. (c) or s.c. (d) were assayed for immunogen IgA responses by ELISA. *X*-axis represents time in weeks, that is, from 0 to 6 weeks. *Y*-axis represents OD @ 450 nm. Statistical analysis was performed using ANOVA and Tukey's mean comparison test. Significance determined by (*) representing *P* < 0.05, (**) *P* < 0.01, and (***) *P* < 0.001.

The purified LTA2B-GH & ntPE-LTA2B-GH fusion proteins, ovalbumin, and TCA peptide were i.n. or s.c. administered to guinea pigs as described in Materials and Methods (Table 1). The earliest serum anti-G-H IgG responses that could be detected by ELISA were observed at week 2 in the animals s.c. immunized with the TCA peptide (Figure 3(b)) and at week 4 in TCA peptide i.n. immunized animals (Figure 3(a)). The animals which received the TCA peptide s.c. produced significant antipeptide IgG responses at weeks 2, 4 and 6, and the animals that received the TCA peptide intranasally produced significant anti-peptide IgG responses at week 4, as compared to other treatment groups. 

 There were no anti-G-H immune responses in the group that received either LTA2B-GH or ntPE-TA2B-GH subcutaneously (Figure 3(b)). However, the group that received LTA2B-GH i.n. induced anti-G-H serum IgG at weeks 4 and 6. This trend was also seen in the group immunized with ntPE-LTA2B-GH intranasally although results were not statistically significant.

 Anti-G-H IgA antibodies in nasal washes were evident at weeks 4 and 6 in guinea pigs receiving ntPE-LTA2B-GH or LTA2B-GH i.n. (Figure 3(c)). However, the group that received the TCA peptide intranasally did not show significant levels of anti-G-H immune responses, indicating that adjuvanticity (in this case derived from the fusion proteins) is essential to elicit mucosal immune responses to the nonreplicating FMDV G-H loop antigen. None of the tested antigens elicited detectable IgA antibody responses in the nasal mucosae of animals immunized subcutaneously (Figure 3(d)). 

### 3.4. Antibody-Secreting Cells (ASCs) in Spleen and Nasal Mucosa

**Figure 4:**
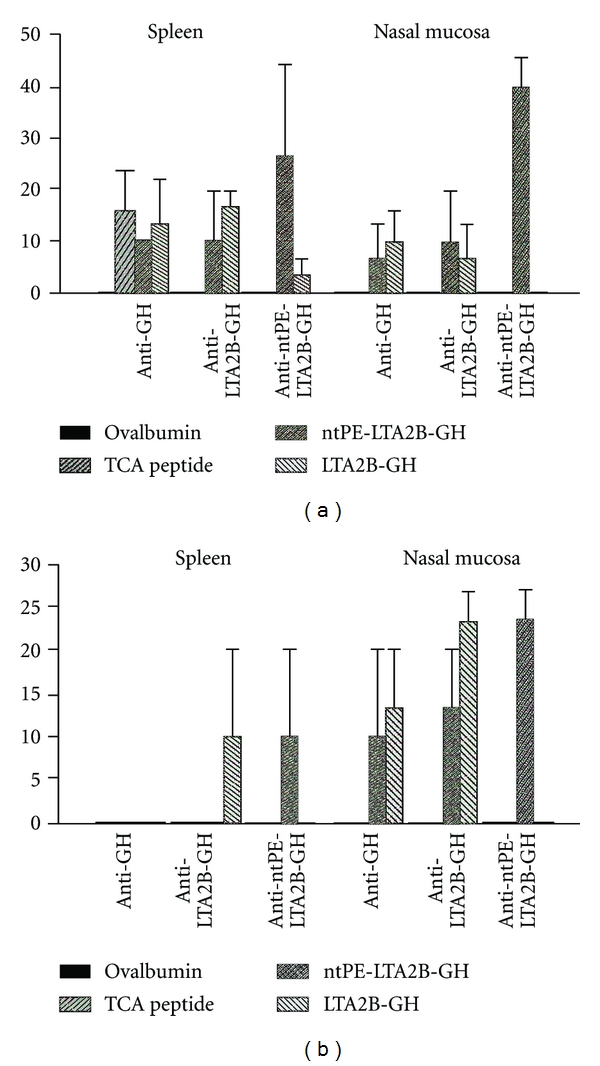
Enumeration of IgG and IgA antibody-secreting cells. Spleen and nasal mucosae were collected from 3 of 6 animals from each group at the conclusion of the study, and lymphocytes were collected from these tissues to enumerate IgG (a) and IgA (b) antibody-secreting cells by ELISPOT. Y-axis represents number of ASC per million cells collected.

At sacrifice, lymphocytes were purified from spleen and nasal mucosae from 3 of 6 animals in each group to enumerate the isotype-specific ASC by ELISPOT. In groups that received the TCA peptide, LTA2B-GH, and ntPE-LTA2B-GH s.c., approximately 10–15 spots/million of anti-TCA peptide IgG lymphocytes were observed in the spleen. In addition, only the groups that received LTA2B-GH and ntPE-LTA2B-GH had an observable number of anti-TCA peptide IgG ASC's in the nasal mucosa (Figure 4(a)), indicating that the presence of a carrier protein and/or adjuvant is necessary for the induction of antibodies against this peptide in the respiratory tissues. Moreover, only the groups that received LTA2B-GH and ntPE-LTA2B-GH i.n. produced IgA ASC specific for the TCA peptide in the nasal mucosa of inoculated animals (Figure 4(b)), again indicating that the adjuvanticity of the fusion proteins is essential to produce mucosal immune responses. No detectable anti-TCA peptide lymphocyte responses could be measured in the spleens of any of the i.n. immunized animals.

## 4. Discussion

The overall goal of this work was to develop mucosal adjuvants that are efficacious in the respiratory tract of animals, with the ultimate goal of inducing immunity to prevent disease, limit viral replication, and prevent the establishment of chronic subclinical infections (namely, carrier animals). Here we provide evidence that a targeted fusion protein based on LTA2B-GH or ntPE-LTA2B-GH elicits mucosal humoral immune responses against an important FMDV VP1 neutralizing epitope in guinea pigs. These fusion proteins were shown to be highly effective mucosal immunogens which induced local antigen-specific antibody formation, including anti-G-H loop IgA in nasal wash samples.

Despite the advantages of eliciting mucosal immune responses to protect animals from infectious diseases, success in this field has been limited due to the induction of low antibody titers, transient immune responses requiring multiple doses of antigen, delayed induction of immune responses, and inefficient establishment of memory responses. Additionally, the stimulation of mucosal immune responses by non-viable antigens is often inefficient and may in some instances result in immunological tolerance [23]. Nonetheless, it is well established that coadministration of adjuvant molecules such as the bacterial toxins CT, LT, PT, and PE secreted by *Vibrio cholerae, E. coli, Bordetella pertussis,* and *Pseudomonas aeruginosa,* respectively, can induce strong mucosal immune responses and prevent the induction of mucosal tolerance [1, 24]. Furthermore, genetic fusion of nontoxic enterotoxins (as carriers) to antigens has been successfully used in vaccination strategies against several pathogens [25–27], and the O_1_ G-H loop fused to CT-B has even been shown to protect from FMDV in a suckling mouse model of infection [28]. Our data are in accordance with these studies while expanding the receptor-binding repertoire of the fusion proteins beyond those of previously designed chimeras. This increases the potential number of species in which any one immunogen may be efficacious while further enhancing mucosal immunity in the respiratory tract of inoculated animals. 

We hypothesized that when the FMDV G-H loop is coupled to the C-terminus of LT-B subunit, a total of five copies will be displayed on a single targeting molecule, as the B subunit of LT forms a pentamer. Also, given that the G-H loop is genetically fused at only one end (LTA2B-GH), it would not likely induce any conformational constraints on the antigen or adjuvant and would properly present multiple copies of the antigen to the immune system. Indeed, efficacy was demonstrated in the i.n. vaccinated groups receiving ntPE-LTA2B-GH or LTA2B-GH (but not the TCA peptide) in terms of antigen-specific secretory IgA production. Others have successfully used similar vaccine/adjuvant approaches, but with alternative fusion sites for the antigen on the enterotoxin. Miyata et al. chemically fused the *Plasmodium vivax* ookinete surface protein, Pvs25, to the N-terminus of CT-B and generated a vaccine construct that induced an immune response which blocked parasite transmission [29]. In another study, the adhesin gene of *Helicobacter pylori* was fused to the A2 subunit of CT (adhesin-CTA2B). This vaccine construct enhanced both IgA and IgG production and protected many mice from infection [30]. While there has been a high degree of success with other enterotoxin/antigen fusion vaccines, we considered another approach for delivery of the antigen, that is, to genetically fuse the FMDV VP1 G-H loop to the C-terminus of LTA2B and replace the toxic A1 domain with the ntPE adjuvant at the N-terminus of the A2 segment (ntPE-LTA2B-GH). Expression of both LTA2B-GH and ntPE on the same plasmid allowed the formation of holotoxin-like chimeras and in this way both the adjuvanticity and carrier properties of LT-B and ntPE could be exploited. Also, given that LT binds a greater diversity of ganglioside receptors than other bacterial toxins, it is likely that the targeting properties of this chimeric protein (consisting of ntPE-LTA2B-GH or LTA2B-GH) has advantageous immunogenic qualities over nonchimeric fusion proteins (although this hypothesis was not empirically studied in this work). This is supported by data indicating that ntPE-LTA2B-GH induced higher amounts of anti-G-H IgA antibodies in nasal wash samples than LTA2B-GH alone, thus indicating that the use of ntPE along with LTA2B has an added effect at inducing immunity against the G-H loop antigen, likely by targeting APC's via binding of CD91 present on their surface.

Purified lymphocytes from spleen and nasal mucosa were restimulated with antigen and groups of animals receiving LTA2B-GH, and ntPE-LTA2B-GH i.n. had the most vigorous mucosal IgG and IgA responses. These results support our central hypothesis that fusion proteins of mucosal adjuvants with nonreplicating antigens are capable of producing mucosal immune responses; unfortunately, neutralization assays performed on the guinea pig sera from week 6 against FMDV indicated that only the group which received the TCA peptide s.c. had high neutralizing titers (data not shown). There may be three possible reasons for this: (a) G-H loop that is conjugated to the C-terminus of the LT-B may not expose immune cells to a neutralizing epitope (possibly due to steric hindrance that might have occurred as a result of the complex nature of the fusion proteins), (b) the presentation of the G-H loop is at an insufficient quantity, or (c) the fusion proteins actually tolerized the animals to the G-H loop antigen. The immunization regime of guinea pigs included 100 *μ*g each of the TCA peptide, ntPE-LTA2B-GH, or LTA2B-GH, and it is noteworthy that the proportion of the G-H loop is only10 *μ*g and 20 *μ*g in ntPE-LTA2B-GH and LTA2B-GH, respectively. An increased dose of ntPE-LTA2B-GH and LTA2B-GH may produce a more robust immune response, but this was not examined in this study. 

 In summary, we have shown that mucosal application of chimeric fusion proteins stimulated the production of antipeptide specific serum IgG and nasal IgA immune responses. At present, it is unclear how ntPE-LTA2B-GH and LTA2B-GH induced the immune responses observed in these studies, whether by facilitating the delivery of five copies of the G-H loop or by enhancing the immune response through the additive effects of these adjuvants, or some combination of both. Taken together, these results are in keeping with studies indicating that genetic fusion of antigens with mucosal carrier/adjuvants is an effective approach for inducing mucosal immunity to nonreplicating antigens.

## Figures and Tables

**Table 1:** Vaccination strategy for guinea pig experiment.

Group	Priming week	Boosting week	Route	Dose	Treatment
I	0	1,2,4	i.n.	100 *μ*g	OVA
II	0	1,2,4	i.n.	100 *μ*g	TCA Peptide
III	0	1,2,4	i.n.	100 *μ*g	LTA2B-GH
IV	0	1,2,4	i.n.	100 *μ*g	ntPE-LTA2B-GH

V	0	3	s.c.	100 *μ*g	OVA
VI	0	3	s.c.	100 *μ*g	TCA Peptide
VII	0	3	s.c.	100 *μ*g	LTA2B-GH
VIII	0	3	s.c.	100 *μ*g	ntPE-LTA2B-GH
